# Impact of the first wave of COVID-19 pandemic on the Hungarian university students’ social and health behaviour

**DOI:** 10.1007/s10389-021-01660-5

**Published:** 2021-10-28

**Authors:** Péter Csépe, Elek Dinya, Péter Balázs, Shahrokh Mirza Hosseini, Gábor Küzdy, László Rosivall

**Affiliations:** 1 Department of Public Health, Semmelweis University, Nagyvárad tér 4, Budapest, 1089 Hungary; 2 Institute of Digital Health Sciences, Semmelweis University, Budapest, Hungary; 3 Avicenna International College, Budapest, Hungary; 4 Institute of Translational Medicine, Semmelweis University, Budapest, Hungary

**Keywords:** COVID-19, Lockdown, Social and health-related behaviour, University students, Hungary

## Abstract

**Background:**

The COVID-19 pandemic brought quick, severe and unexpected changes to our everyday life and also changed the traditional education pattern of Semmelweis University in the middle of academic year 2019–2020. We explored adaptive changes in Hungarian students’ behaviour and their time-budget in order to determine whether quarantine and/or fear of infection were responsible for these changes.

**Methods:**

A self-administered online questionnaire was distributed to all students in the Hungarian language program (*N* = 7436) of Semmelweis University. Information was collected on basic demographic data, knowledge and attitude about COVID-19, methods of prevention as well as the students’ behaviour before, during and after the first wave of the pandemic. Statistical analyses were processed using the IBM-SPSS 25.0 software package.

**Results:**

The overall response rate was 11% (*N* = 816). Only complete responses were processed (55%, *N* = 447). Among these responders, 83% did not fear the pandemic. Those who greatly feared COVID-19 infection strictly kept all regulations. The number of non-smokers increased by the end of the first wave. The nutrition of 100 students (21%) became healthier and the lockdown reduced the level of physical activity.

**Conclusion:**

Social and health-related behaviour of medical students changed basically during the first wave of the pandemic and some changes remained after it in tobacco smoking, nutrition and sleeping habits. Time-budget of students changed significantly during the pandemic and did not return to the baseline values. Results of this study justify future multiple systematic research to analyse and better understand the short- and long-term effects of the current crisis.

## Introduction

The ongoing COVID-19 pandemic has left remarkable impacts on all countries including Hungary. On 4 March 2020, Hungary’s Prime Minister officially announced the first two cases of coronavirus infection detected among university students in the country (Ritchie et al. 2020). Initially, the academic calendar was not revised, but the affected students and their contacts could not attend the lectures and practices. Semmelweis University, as one of Hungary’s leading universities, introduced additional precautionary measures such as social distancing, self-isolation and quarantine in order to minimize the spread of the virus (de Souza Melo et al. 2021). These changes were implemented in the middle of the academic year 2019–2020 with enormous impact on teaching, learning and the students’ behaviour. Hungary’s Chief Medical Officer declared the end of the first wave of the COVID-19 pandemic 16 June 2020. Nevertheless, e-learning and online exams remained operative, which inevitably prevented the students from visiting the university’s facilities. Hungary has for decades not experienced the spread of deadly infectious diseases owing to the extremely high vaccination rate and high level of hygiene. Thus, the current generation of students has never experienced any kind of lockdown. The COVID-19 pandemic has had a massive impact on human health and social relations with mandated sudden lifestyle changes through social distancing, face and nose mask wearing and at-home isolation. The aim of this study was to explore COVID-19 related knowledge, attitude and behaviour (smoking, physical activity, nutrition) of students at Semmelweis University. We collected data on time-budget (leisure-time, working/learning time) as well. Additional information was collected on epidemiological data, means of prevention and sources of information, the influence of the pandemic on individuals and communities, especially due to restrictive authority actions taken to control the pandemic. The students also reported their fear concerning possible infection.

## Methods

We developed a special self-administered questionnaire tailored to the aims of this study. Information was gathered relevant to the pandemic and related rules and regulations. Preliminary validation was processed in a representative group of ten medical students followed by in-depth interviews. Then a pilot survey was conducted and evaluated by appropriate statistical analysis. The internal consistency was measured with Cronbach’s alpha (= 0.685). Participants suggested several changes in wording to clarify some questions.

The final version of our questionnaire contained 50 closed items, which explored basic demographic data, knowledge about COVID-19, methods of preventive measures, pandemic impact on the participants’ behaviour and lifestyle as well as effects of national and local social restrictions. Health-related behavioural changes have also been investigated with questions on smoking, nutrition and physical activity patterns before, during and after the first wave of the COVID-19 pandemic. Data on time-budget, including time for leisure activities, working, learning and sleeping were also collected in the same time frame. The students’ knowledge about COVID-19, preventive methods and the level of fear of infection were also examined as factors with potential impact on health-related behaviour. Information on physical activity (PA) was collected via questions referring to the time spent on PA before, during and after the first wave of the COVID-19 pandemic. Specific questions were: ‘How much time did you usually spend with physical activities before and after the pandemic?’ and ‘How did you change your PA during the pandemic?’ The participants answered the questions in approximately 15 min on average.

We distributed the questionnaire to all students in the Hungarian language program of all faculties of Semmelweis University (*n* = 7436) through the university’s online educational administration system (NEPTUN). The survey started just after the first wave of COVID-19, on 29 June 2020. At that time the number of positive tested cases was six per day in Hungary, while there were 4145 active cases nationwide, and practically no restrictive measures were in effect. Because it was not possible to conduct serial surveys, students estimated behavioural and timeline changes on their recall; thus, relevant bias must be taken into account.

Results are expressed as mean and standard deviation of the mean (SD). The distribution pattern of data was checked by the Shapiro–Wilk’s test. When non-normal data could not be rejected, homogeneity of variances was assessed via the Levene’s test. Means were compared by main effects analysis of variance (ANOVA) with Tukey’s correction for multiple comparisons applied where appropriate. Pearson chi-square test (χ^2^) or Fisher’s exact test was applied to sets of categorical data. The analysis was two-sided, with a level of significance of α = 0.05. All statistical analyses were done using the IBM-SPSS v. 25.0 (SPSS Inc., Chicago, IL) software package.

## Results

There were 7436 registered Hungarian students (females 5560, males 1876) at six faculties (medicine, dentistry, pharmacy, health sciences, public sciences and conductive education) of Semmelweis University. For analyses, we collapsed the data of the last three faculties. An overwhelming majority of participants administered the questionnaire between 29 June and 5 July 2020. The overall response rate was 11% (*n* = 816) and female students outnumbered the males (75.9 v. 24.1%) similar to the target population (74.8 v. 25.2%). In other words, the female/male ratio of the target population was 2.96/1 (5560/1876) and that of the final sample 3.03/1 (336/111) and the difference was not significant.

The responders’ mean age was 25.6 ± 7.2 years and that of females was significantly higher than males (*p* = 0.006). Because numerous forms were incomplete concerning fear of coronavirus infection (*n* = 369), smoking (*n* = 370) and eating (n = 369) habits, and physical activity (n = 370) only the complete forms were used for further analyses.

One third of responders lived positively through the lockdown, while another one third experienced this period in a negative way, and the last third did not indicate any opinion. A high percentage of students (83%) did not fear the pandemic (not at all) or just a little. Significant gender differences were not indicated (by Pearson chi-square test), nor could we find any significant differences (*p* = 0.470) among students of specific faculties. Within the collapsed category ‘I am afraid’ (*n* = 77) only 28 (3.4%) of the whole sample admitted they lived in permanent fear during the first wave of the pandemic. The regulations had been observed ‘always’ or ‘yes, most of the time’ (*n* = 431) by 97.1% (males = 102, females = 329) with no significant difference if compared to the target population (*p* = 0.47). Table 1 shows that there was strong association between the level of fear and observing the lockdown rules. Considering observation of regulations related to the level of fear, there were statistically significant (*p* < 0.001) differences among groups who answered about observing the regulations as ‘yes most of the time’ compared to those who answered ‘no most of the time’. There was also a significant difference in observing the regulations among groups who reported ‘a little fear’ and groups who reported ‘I am afraid’.

**Table 1:** Percent distribution of fear and adherence to rules of students (absolute values are in brackets)

		Were you afraid of coronavirus infection?			
		Not at all	A little	I am afraid	Total
Have you complied with the regulations concerning the coronavirus pandemic?	Always, all specifications	21 (42)	53 (107)	27 (54)	100 (203)
	Yes, most of the time	32 (74)	58 (135)	10 (22)	100 (231)
	No, most of the time	54 (7)	38 (5)	8 (1)	100 (13)
	Total	28 (123)	55 (247)	17 (77)	100 (447)

The chi-square statistic is 28.7519, df = 4. The *p* value is <0.0001. The result is significant at *p* < 0.05

Concerning the tobacco smoking status of students (Table 2), 9.6% (*n* = 39) smoked regularly before the pandemic (i.e. smoked at least 1 cigarette a day). Smoking habits differed significantly among students of specific faculties (*p* = 0.049). Only one student was a heavy smoker (>10 cigarettes a day), while 75.2% (*n* = 304) were non-smokers (never-smokers or previously quitted). Among females (*n* = 334) non-smokers outnumbered significantly (82.7 v. 27.7%) the males (*n* = 112).

**Table 2:** Smoking status of students prior to the first wave of the pandemic in specific faculties (absolute values are in brackets)

	Smoking habits			
Faculty	I never smoked	Occasionally	More than 10 cigarette a day	Total
Medicine	68 (132)	27 (54)	4 (8)	100 (194)
Dentistry	80 (28)	14 (5)	5 (2)	100 (35)
Pharmacy	78 (36)	17 (8)	4 (2)	100 (46)
Collapsed	79 (136)	13 (23)	7 (13)	100 (172)
Total	74 (332)	20 (90)	5 (25)	100 (447)

The chi-square statistic is 14.2519, df = 6. The *p* value is .026945. The result is significant at *p* < 0.05

After the lockdown and first wave of the pandemic the number of occasional smokers (males 23, females 44) decreased from 67 to 9 (males 4 females 5) with non-significant (*p* = 0.551) gender difference. There were 21 students (males 10, females 11) smoking at least 1 cigarette a day and their number increased to 50 (males 24, females 26) that of smoking ≤10 cigarette a day decreased from 22 (males 3 females 13) to 14 (males 3 females 11) and 3 heavy smokers did not change their habit.

Concerning PA there were no significant differences among the faculties. Only 49 students (11%) reported (PA) a sufficient level (more than 30 min a day) before the pandemic, while 249 did not exercise at all or just occasionally (1–2 times) weekly. No significant differences were recognized between males and females any time (*p* = 0.291, *p* = 0.139, *p* = 0.839, respectively). During the lockdown 230 students reported less PA, while 140 more, but there was no change in PA habits of 107 cases. After the first wave of the pandemic, 84 students reported minimal PA or just 1–2 times weekly, while the number of sufficient (30 min daily) PA increased to 48 (13%).

In dietary habits there were no significant differences as students of different faculties were compared before, during and after the first wave of the pandemic. Before the pandemic, 237 students (190 females, 47 males) considered their nutrition habits as ‘healthy’ indicating that they always or in most cases followed the principles of healthy eating. The gender difference was significant (*p* = 0.005). In contrast, 47 responders did not follow these principles most of the time. The number of answers ‘sometimes yes, sometimes no’ was 163. Table 3 shows that the nutrition of 99 students (77 females) changed towards healthier during the first wave of the pandemic, while 87 students (65 females) reported a decline in health standards of their dietary habits. Meanwhile, 261 students (197 females, 64 males) reported no changes. No significant gender differences (*p* = 0.984) were detected. As to the item ‘Do you eat in a healthy way now’ (after the pandemic) 41 students (24 females, 17 males) said they did not follow the principles of healthy eating, while 249 students (195 females, 54 males) followed them. The number of females was significantly (*p* = 0.006) higher.

**Table 3:** Did your eating habits change during the pandemic (absolute values in brackets)

Faculty	I ate healthier	I ate essentially the same way	I ate less healthy	Total
Medicine	24% (46)	56% (109)	20% (39)	100% (194)
Dentistry	17% (6)	60% (21)	23% (8)	100% (35)
Pharmacy	24% (11)	54% (25)	22% (10)	100% (46)
Collapsed	21% (36)	62% (106)	17% (30)	100% (172)
Total	22% (99)	58% (261)	19% (87)	100% (447)

The chi-square statistic is 2.1358, df = 6. The *p* value is .90679. The result is not significant at *p* < 0.05

Table 4 shows the students’ time (in hour) spent on sleeping, working/studying and recreation (fun and leisure) before, during and after the pandemic. We considered the time spent for sleep before the pandemic as the baseline (7.16 ± 2.33 h). Sleep period increased during the pandemic (8.54 ± 3.29) and decreased after the first wave; however, it did not return to the values before the pandemic (8.65 ± 1.22 h) when compared to the baseline (3.24 ± 2.45 h). Both differences are significant at *p* < 0.001 level.

**Table 4:** Descriptive statistical data of time (in hour) spent on work: before, during and after the pandemic

			Pandemic		
			Before	During	After
How many hours a day did you spend during the pandemic on:	Faculty	N	Mean	Mean	Mean
Sleeping	Medicine	194	7.21 ± 1.04	8.43 ± 1.53	7.42 ± 1.1
	Dentistry	35	7.31 ± 0.99	9.06 ± 1.37	8.06 ± 1.06
	Pharmacy	46	7.24 ± 1.02	8.65 ± 1.46	8.11 ± 1.38
	Collapsed faculties	172	7.06 ± 1.01	8.52 ± 1.74	7.69 ± 1.27
Work/study	Medicine	194	10.16 ± 2.17	7.8 ± 3.10	8.8 ± 3.42
	Dentistry	35	11.09 ± 2.10	7.91 ± 2.57	6.86 ± 3.97
	Pharmacy	46	10.74 ± 2.33	7.76 ± 3.26	6.54 ± 3.31
	Collapsed faculties	172	10.42 ± 2.53	7.44 ± 3.62	7.79 ± 3.77
Fun and leisure	Medicine	194	3.57 ± 1.63	3.93 ± 2.46	3.46 ± 2.31
	Dentistry	35	3.17 ± 1.62	4.0 ± 1.86	4.89 ± 2.89
	Pharmacy	46	2.93 ± 1.38	3.93 ± 2.8	4.67 ± 2.81
	Collapsed faculties	172	2.97 ± 1.60	3.48 ± 2.25	3.55 ± 2.30

We considered the time students spent for working and studying before the pandemic as a baseline (10.40 ± 2.33). This time decreased during the first wave of pandemic (7.66 ± 3.28) and increased after; however, it did not reach the time before the first wave of the pandemic (8.03 ± 3.67) when compared to the baseline. Both these differences were significant (*p* < 0.001 and *p* = 0.001, respectively). No significant difference in working and studying time was measured during and after the first wave of the pandemic. Both differences are significant (*p* = 0.041 and *p* < 0.001 respectively).

We considered the time students spent for recreation before the pandemic as the baseline (3.24 ± 1.62 h). Recreation time increased during the pandemic (3.76 ± 2.38 h) and remained higher (3.73 ± 2.38) compared to the baseline. Both differences are significant (p < 0.001 and *p* = 0.001, respectively).

## Discussion

Pandemics on the present wide scale are rare; hence, research on their psychological/behavioural effects are pre-eminently important. Recently, two pandemics had emerged: the SARS outbreak in 2003 and H1N1 outbreaks in 2009 (Steel Fisher et al. 2010). By the summer of 2003 the acute psychological impact of SARS had widely been studied. Significant emotional distress was present in 18–57% of the population due to the quarantine, fear of contagion, concern for family, job stress and interpersonal isolation. Papers with similar results have been published related to the H1N1 pandemic. Reports on the effects of restrictive measures on human behaviour during the current pandemic are growing rapidly (Zhou et al. 2020).

In the present pandemic, Hungary’s cumulative number of infections was 4145 by the end of June, one of the lowest in Europe. In the survey period only 4–6patients/day were diagnosed with COVID-19 due to the strict public health law enforcement. This study used a well-designed questionnaire, which was distributed just after some social relaxations following the first lock-down period of the pandemic. This way we succeeded to evaluate the behaviour of students before, during and after the first wave of the COVID-19 pandemic. Response rate and the number of completed questionnaires were lower than expected, partly because questionnaires were distributed just after the university examination period. Another possible explanation was the students’ low interest towards the first wave of the COVID-19 pandemic. Nevertheless, our findings should be considered as representative because the female/male ratio of our sample did not differ significantly from that of the whole target population.

Fear is an adaptive response to the emerging danger. Constant and prolonged fear can lead to modified behavioural patterns and even mental or psychosomatic dysfunctions can influence the population’s protective behaviour. The coronavirus pandemic frequently causes fear, as its immediate consequences for the public have produced unprecedented challenges for the education and healthcare systems (Hiep et al. 2020; Hosen et al. 2021). National polls indicated sudden increases in fear and worries related to the virus in many countries (Asmundson and Taylor 2020). The quick spread of the coronavirus in Europe, the visual media coverage of the events and hospital disturbances caused a widespread panic among the general population. Counterbalancing it, during the first wave of the pandemic and the successive lockdown, major health-related media messages and organized advertisements were targeted to raise awareness about ways of reducing the risk of infection such as social distancing, washing hands and wearing a face–nose mask. The overwhelming majority of Hungarian students participating in our study did not mention any serious fear of the COVID-19 pandemic. However, it remains to be investigated whether a similar attitude towards the COVID-19 pandemic fear can be detected in general university student communities other than medical/health students.

Sudden widespread and restrictive measures with a long period of lock-down changed the health-related behaviours. Indeed, with large segments of the population under conditions of isolation, modifications to lifestyle-related behaviours are largely inevitable. These are likely to include changes in sleep pattern, alcohol consumption, physical activity and dietary habits (Arora and Grey 2020). The effects of lockdown on smoking habits were not unambiguous. According to an Italian study, smokers smoked considerably more during the lockdown period. The main concern for increasing cigarette consumption was due to an increased mental distress (Carreras, et al. 2021). Other studies have shown no significant decrease in smoking prevalence. Nevertheless, COVID-19 was associated with increases in quit attempts and successful cessation among past year smokers (Jackson et al. 2020). According to a four country survey (Australia, Canada, England and United States) smokers were more likely to quit or reduce their habit if they had greater fear about COVID-19(Gravely et al. 2021).

In our study, we can say there is no correlation between changes in smoking habits and the fear of COVID-19 infection despite the well-known fact that smoking is a risk factor for severity of COVID-19. This indicates that extreme social measures taken against the spreading of the virus are in the background of changes in smoking habits rather than the pandemic effect on health itself. Remarkably, lockdown did not significantly instigate smoking because some students smoked less and some quit smoking after the first wave of COVID-19, in order to follow a healthier lifestyle as a protection against the pandemic (Table 2).

Physical activity has been identified as an important protective factor reducing the risk of numerous somatic and mental disorders (Mammen and Faulkner 2013). It is reasonable to assume that lockdown leads to reduced levels of physical activity in the general population (Füzéki et al. 2020). Because in our study there was no significant difference in recreation time during and after the first wave of pandemic, we can assume that students maintained their recreation habits after the first wave of the pandemic.

Compelling evidence showed that dietary habits are affected by conditions of stress, distress and emotional disturbances. Changes in dietary patterns during the outbreak of COVID-19 could also be driven by fear and anxiety. Since the outbreak of COVID-19, there have been multiple media reports of panic buying and stockpiling of household wares. If consumers are buying more food and raw materials for cooking and baking, they may be preparing more home meals. Thus, the effect of the COVID-19 lockdown both negatively and positively impacted dietary practices throughout the world. An increase in fresh produce and home cooking may reduce the consumption of comfort food, while an increase of snacks consumed and more frequent meals may have a negative impact during quarantine, especially if associated with weight gain and limited physical activity (Bennett et al. 2021). We assumed Hungarian students also prepared more meals at home; however, their diet did not change positively.

Sleep is absolutely fundamental for optimal health (von Ruesten et al. 2012). Social isolation and lockdown may be the silver lining to COVID-19 as it permits most individuals more time at home, thus allowing for some flexibility in sleep–wake times and sleep duration. Students postponed bed and wake-up times and chronotype shifted towards eveningness. Sleep duration increased and quality of sleep improved only among students who had shorter sleep duration prior to the pandemic (Genta et al. 2021). Students home-schooled during lockdown can keep their own schedule because they are not required to attend live class sessions early in the morning. Thus, there may be positive downstream effects on a range of cognitive outcomes (Walker 2008, Rodríguez-Almagro et al. 2020). A general lockdown, self-isolation and social distancing has resulted in significant changes in the daily routines and lifestyle in terms of healthier or unhealthier behaviour (van der Werf et al. 2021).

University students are most likely to benefit from lockdown with respect to sleep (Wright et al. 2020). Restrictions reduced the mismatch between external (social) and internal (biological)sleep–wake timing, as indexed by significant reductions in social jetlag and social sleep restriction, with a concomitant increase in sleep duration (Blume et al. 2020). The students’ time spent on sleep, leisure, work and computer use showed significant gender specificity. Social restrictions opened the window of opportunity for some students to develop and maintain healthy and consistent sleeping habits if they prioritized them at all. Time-budget of students changed significantly during the pandemic and did not return to the baseline before the pandemic.

We did not find any other similar surveys exploring the period immediately after the first wave. Our results show a unique overall picture about the effects of the COVID-19 pandemic and related restrictive measures on the behavioural change of university students. Systematic research is needed to understand better the short- and long-term outcomes of the current crisis affecting individual or group behavioural changes and their persisting nature.

## Data Availability

Data are available upon request.

## Code availability

N/A
